# Bacteriophage-based nano-biosensors for the fast impedimetric determination of pathogens in food samples

**DOI:** 10.1038/s41598-023-30520-3

**Published:** 2023-03-01

**Authors:** Nader Abdelhamied, Fatma Abdelrahman, Ayman El-Shibiny, Rabeay Y. A. Hassan

**Affiliations:** 1 Nanoscience Program, University of Science and Technology (UST), Zewail City of Science and Technology, 6Th October City, Giza, 12578 Egypt; 2 Center for Microbiology and Phage Therapy, Zewail City of Science and Technology, Giza, 12578 Egypt

**Keywords:** Analytical biochemistry, Sensors and probes

## Abstract

The early and rapid detection of pathogenic microorganisms is of critical importance in addressing serious public health issues. Here, a new bacteriophage-based nano-biosensor was constructed and the electrochemical impedimetric method was fully optimized and applied for the quantitative detection of *Escherichia coli* O157:H7 in food samples. The impact of using a nanocomposite consisting of gold nanoparticles (AuNPs), multi-walled carbon nanotubes (MWCNTs), and tungsten oxide nanostructures (WO_3_) on the electrochemical performance of disposable screen printed electrodes was identified using the cyclic voltammetry and electrochemical impedance spectroscopy. The use nanomaterials enabled high capturing sensitivity against the targeting bacterial host cells with the limit of detection of 3.0 CFU/ml. Moreover, selectivity of the covalently immobilized active phage was tested against several non-targeting bacterial strains, where a high specificity was achieved. Thus, the targeting foodborne pathogen was successfully detected in food samples with high specificity, and the sensor provided an excellent recovery rate ranging from 90.0 to 108%. Accordingly, the newly developed phage-biosensor is recommended as a disposable label-free impedimetric biosensor for the quick and real-time monitoring of food quality.

## Introduction

Access to sufficient amounts of clean, safe and nutritious food products is the key for promoting good health, and sustaining human-life. Thus, unsafe, and contaminated food containing pathogenic bacteria, viruses, parasites or harmful chemical substances are the main reason for spreading more than 200 common diseases, ranging from diarrhea to cancers^1^. Enterohaemorrhagic *Escherichia coli* (EHEC), *Campylobacter* and *Salmonella*, are the most common foodborne pathogens that affect millions of people annually, with severe and fatal outcomes^2^. *E. coli* is a fecal coliform Gram-negative bacterium that is found in the intestines of birds, mammals, and human gut. Most strains of *E. coli* are typically harmless^3^. However, pathogenic *E. coli* strains are classified into 6 groups; enterohemorrhagic *E. coli*, diffusely adherent *E. coli*, enteroaggregative *E. coli*, enteroinvasive *E. coli*, enteropathogenic *E. coli*, and enterotoxigenic *E. coli*^4^. *E. coli* O157:H7 is considered as the most important enterohemorrhagic bacterial strain because of its ability to produce lethal Hemolytic Uremic Syndrome (HUS), and to produce Shiga-toxins that cause severe health problems. Infection with the *E. coli* mostly occurred through the consumption of water, milk, food, meat, and vegetables that are contaminated with fecal sources^5^. Early and rapid diagnosis is necessary for preventing or decreasing the serious infection caused by food contamination. Therefore, the development of a sensitive, rapid, selective, accurate, and easy-to-use detection approach of *E. coli* O157:H7 is a must^6,7^. Generally, traditional microbiological methods for the detection of bacteria include pre and selective enrichment, serological confirmation, and biochemical screening. These methods are time-consuming, vague in terms of results, and laborious^8,9^. Polymerase chain reaction (PCR), enzyme-linked immunosorbent assay (ELISA), and plate culture are the typical methods that are currently used for *E. coli* O157:H7 detection in food and clinical samples^10,11^. These techniques require complex sample preparation steps, such as intracellular extraction followed by complicated steps of amplification and purification. These challenges and drawbacks make such molecular techniques a complicated approach, as they require highly trained personnel and highly expensive lab-based instruments. Furthermore, these techniques have not yet been widely used for on-site detection, and they are not adopted in commercial diagnostic laboratories. Accordingly, designing disposable, portable, label-free, and reliable diagnostic platforms for the rapid and onsite bacterial contamination in real food samples is still needed^9,12,13^.

To that end, biosensors attracted the attention of the scientific community due to their high selectivity, sensitivity and accuracy for the fast determination of microbial contamination and the biological activities of pathogens^6,14,15^. Biosensors are analytical devices that use biochemical/biological reactions to detect a single or multiple targeting analytes^16,17^. A typical biosensor consists of three main components, the first is the recognition element(s) (e.g. antibodies, DNA, enzymes, bacteriophages, cells, aptamers, or biomimetic (artificial) sensing materials)) which specifically reacts with a target molecule^8,15^.

Biosensors global markets are consolidating due to the growing popularity of medical equipment and tailored medications, increased preference for disposable and non-invasive biosensors, and supported the research collaboration and agreements between diverse manufacturers, and academic research institutions. Accordingly, the forecast of the global biosensor market size was valued at 25 Billion US Dollars in 2022 and is expected to expand at a compound annual growth rate of 8.0% from 2022 to 2030. As the biosensor has great privileges in the biomarker diseases and diagnosis of infection, plenty of biosensing technologies will be commercially available in the market. Optical and electrochemical biosensors are the most common techniques used for the fabrication of point-of-care devices (POC) for quantitative analysis of biomarkers as well as for infectious diseases^15,18^.

High-affinity recognition elements are needed for the high selectivity and biosensing specificity, thus the selection of such biorecognition elements is very critical. In this regard, antibody-based biosensors (immunosensors) may challenge a cross-reactivity with the unrelated targets that have similar antigenic structures and lead to false-positive signals^15,19,20^. Aptamer-based biosensors offered several advantages over immunosensors, hence they are particularly suited with small-sized molecules. However, they still face the challenges to be used for the detection of large organisms such as bacteria. Moreover, both aptamers and antibodies are not able to identify the biological activity, and cell viability of microorganisms^21^.

Interestingly, phages or bacteriophages provide natural affinity to their host bacteria cells, thus they can serve as high efficient bioreceptors for the development of electrochemical biosensing platforms. Phage is a virus that can specifically infect (kill) a selected bacteria. It binds itself only to a susceptible bacterium and injects its DNA into the host cell. Accordingly, new phages assemble and burst out of the bacterium in the cell lysis process^22^. Therefore, phage-sensing platforms have high specificity against their bacterial hosts, and they have high thermal stability, and are not affected by the changes in surrounding compositions^23,24^.

On the other hand, nanomaterials been extensively used in the fabrication of electrochemical nano-biosensors to enable high electro-catalytic activity, electrical conductivity, and to support the orientation and stability of the bio-recognition element(s)^25–28^. Herein, a nanostructured T4-like phage-impedimetric biosensor is designed and fabricated with a novel nanocomposite substrate to detect *E. coli* O157:H7 in food samples.

## Results and discussion

### Impact of nanomaterials on the sensors performance

Being a label-free, highly sensitive, and not affected by the presence of turbid or colored components in complex sample matrices, impedimetric techniques are implemented here for constructing a robust phage-based nanosensor for the fast bacterial recognition. Bacteriophage (phage ZCEC5) is selected as the active bio-recognition element due to its high sensitivity and selectivity for the detection of whole cells of *E. coli* O157:H7.

Basically, phages are highly specific to their bacterial host where each phage can only recognize special receptors in its specific host to infect it and produce a new progeny. This process is called a phage-bacteria communication mechanism that governs the phage lysis decision, which is mainly dependent on phage-host interactions. This means that the assigned phage (Phage ZCEC5) can be used to selectively identify and detect the targeting food-borne pathogen.

Designing a high-performance electrochemical biosensor is usually achieved when a highly conductive, high electroactive, and expandable surface area of the sensor is offered. Therefore, selection of a proper sensor platform is necessary to enhance the reproducibility of the impedimetric signal, to expand the sensing surface area, and to support the bacteriophage immobilization. Additionally, sensor materials have a significant influence on the kinetics of the redox reactions taking place at the interfaces, and thus they support the success of electrochemical processes. Herein, a nanocomposite consisting of gold nanoparticles (Au NPs), multi-walled carbon nanotubes (MWCNTs), and tungsten oxide nanostructures (WO_3_) was implemented as the sensor platform. The disposable sensor’s chips were modified with the nanocomposite, or its individual constituents. Previously, we constructed a double-mediated impedimetric viral biosensor for the rapid detection of the whole SARS-CoV-2 particles, whereas the nanocomposite (WO_3_/MWCNTs) was exploited for enlarging the imprinted surface area^29^.

**Figure 1:**
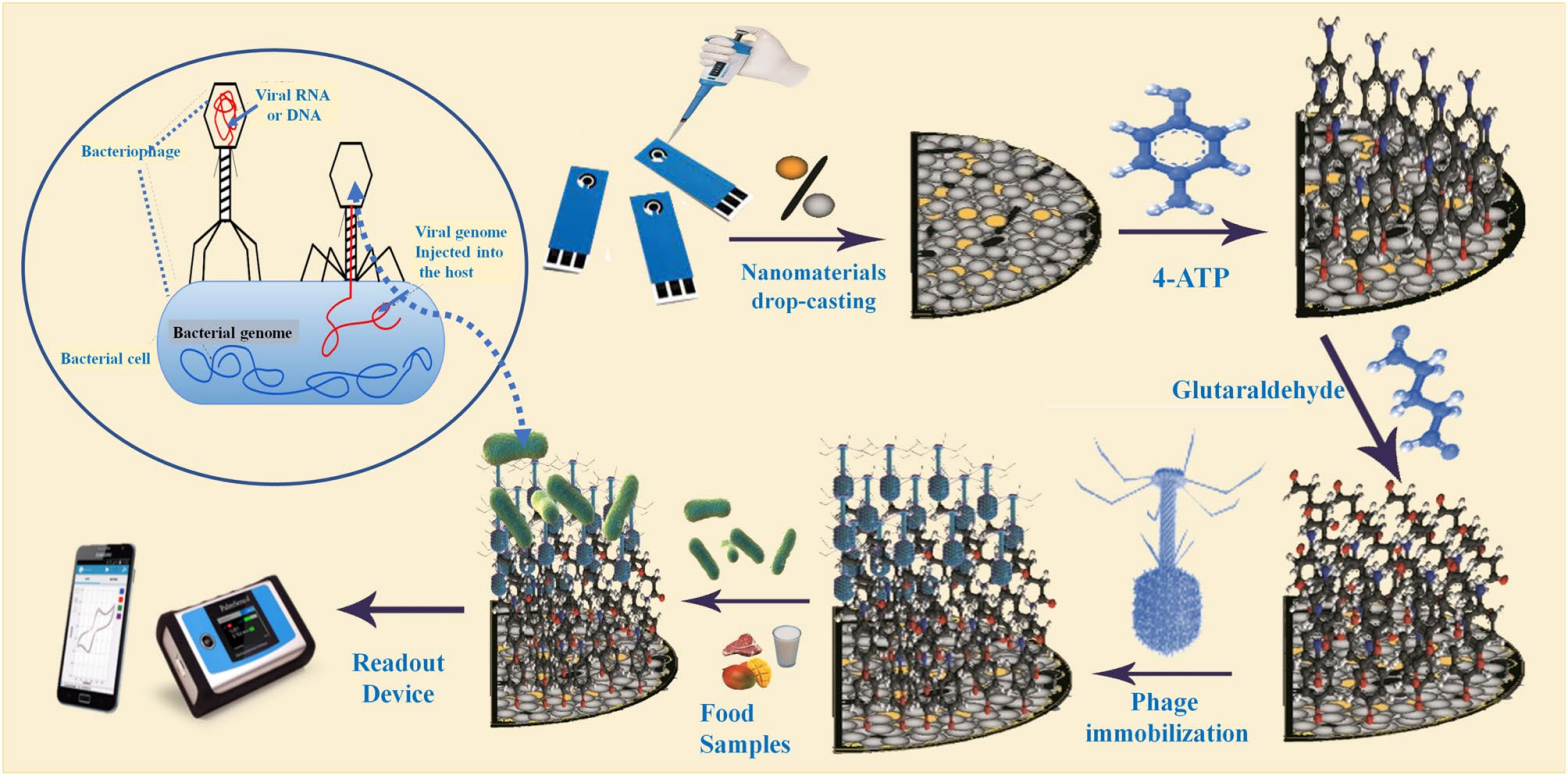
Subsequent process of the fabrication of the phage-based biosensing approach for the rapid impedimetric detection of *E. coli*. Firstly, the electrode surface modification with nanomaterials was carried out before forming a self-assembled monolayer of 4-ATP as the main cross-linker. Then, surface activation by glutaraldehyde solution (2%) was conducted before the immobilization of the targeting bacteriophage. Eventually, applications on food sample analysis were performed using the PalmSens-4 portable potentiostat.

**Figure 2:**
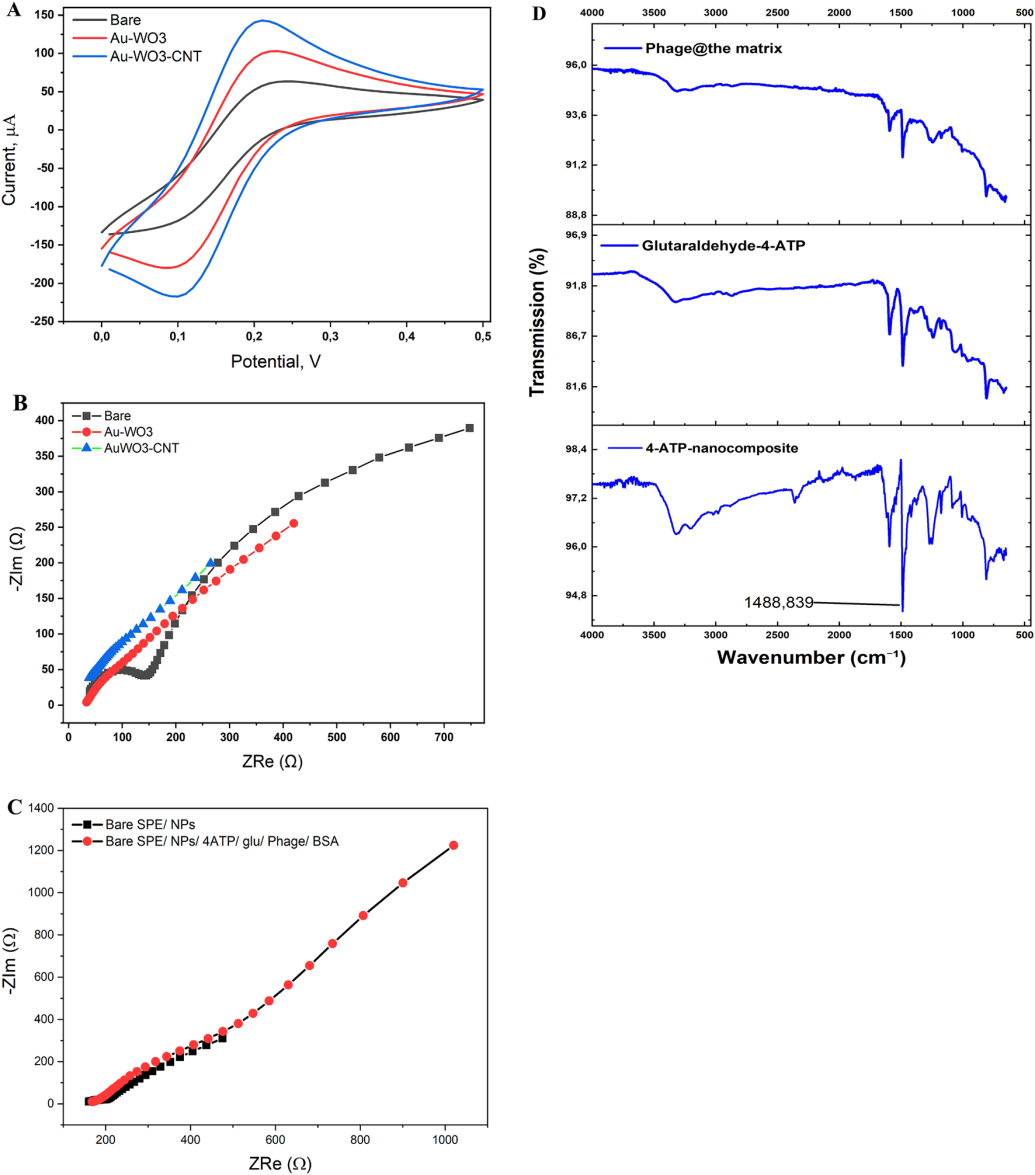
(**A**) Cyclic voltammetric responses of electrode surfaces towards the redox reactions of Ferricyanide (FCN, 5 mM) as the standard redox probe. (**B**) Impedimetric responses of electrode surfaces towards the redox reaction of Ferricyanide (FCN, 5 mM) as the standard redox probe. (**C**) Impedimetric responses of electrode surfaces before and after the immobilization of the active bacteriophage. (**D**) FTIR spectra of the preparation steps of the phage-based nano-biosensor.

For the effective covalent immobilization of the active phage onto the sensor surface modified with the nanomaterials, 4-aminothiophenol (4-ATP) was self-assembled onto the nanostructured surface, followed by the activation with glutaraldehyde chain to form a well oriented self-assembled monolayer, as shown in Fig. 1. Consequently, electrochemical characterizations (shown in Fig. 2A and B) were conducted, where the cyclic voltammetric (CV) and electrochemical impedance spectroscopic (EIS) measurements showed that the incorporation of the nanocomposite led to synergetic electrochemical enhancements (increases in the rates of the oxidation–reduction reactions of the standard redox probe) due to the high conductivity, and electrocatalytic activity and the expansion in the surface area acquired by the tungsten oxides and carbon nanotubes. Similarly, the EIS data confirmed the importance of the nanocomposite for decreasing the impedimetric signals due to the acceleration of electron transfer from the solution interface to the electrode surface.

Additionally, the use of the CV and EIS was very important to characterize the stability and reactivity of the chemically conjugated active phage into the modified surface with the nanocomposite. In terms of that, a strong increase in the EIS response was obtained after cross-linking the phage through the covalent binding with the 4-ATP/glutaraldehyde chain (As shown in Fig. 2C). The increase in the EIS readouts is attributed to the fact that the active site for sensing the redox reaction on the electrode surface has been blocked as a result of the phage immobilization and bacterial capturing.

For the functional analysis, Fourier-Transform Infrared (FTIR) spectra of the sensor components were investigated. The chemical cross-linking using the 4-ATP/glutaraldehyde which was made for the effective covalent immobilization of the T4-like phage (ZCEC5) on the nano-structured surfaces was identified whereas a very strong sharp peak at 1489 cm^-1^ combined with another broad peak was obtained at 3300 cm^-1^ which are corresponding to the characteristic peaks for the stretching vibrations of (-NH_2_) that representing the successful formation of the self-assembly of 4-aminothiophenol (4-ATP) onto the nanostructures electrode surface (nanocomposite-4-ATP SAM)^30^. Consequently, when the ZCEC5 was covalently immobilized onto the glutaraldehyde-activated 4-ATP surface, a decrease in the stretching vibrations of the (-NH_2_) was observed indicating the chemical attachments of the phage into the functionalized sensor surface, Fig. 2D.

### Double-mediated biosensing system

**Figure 3:**
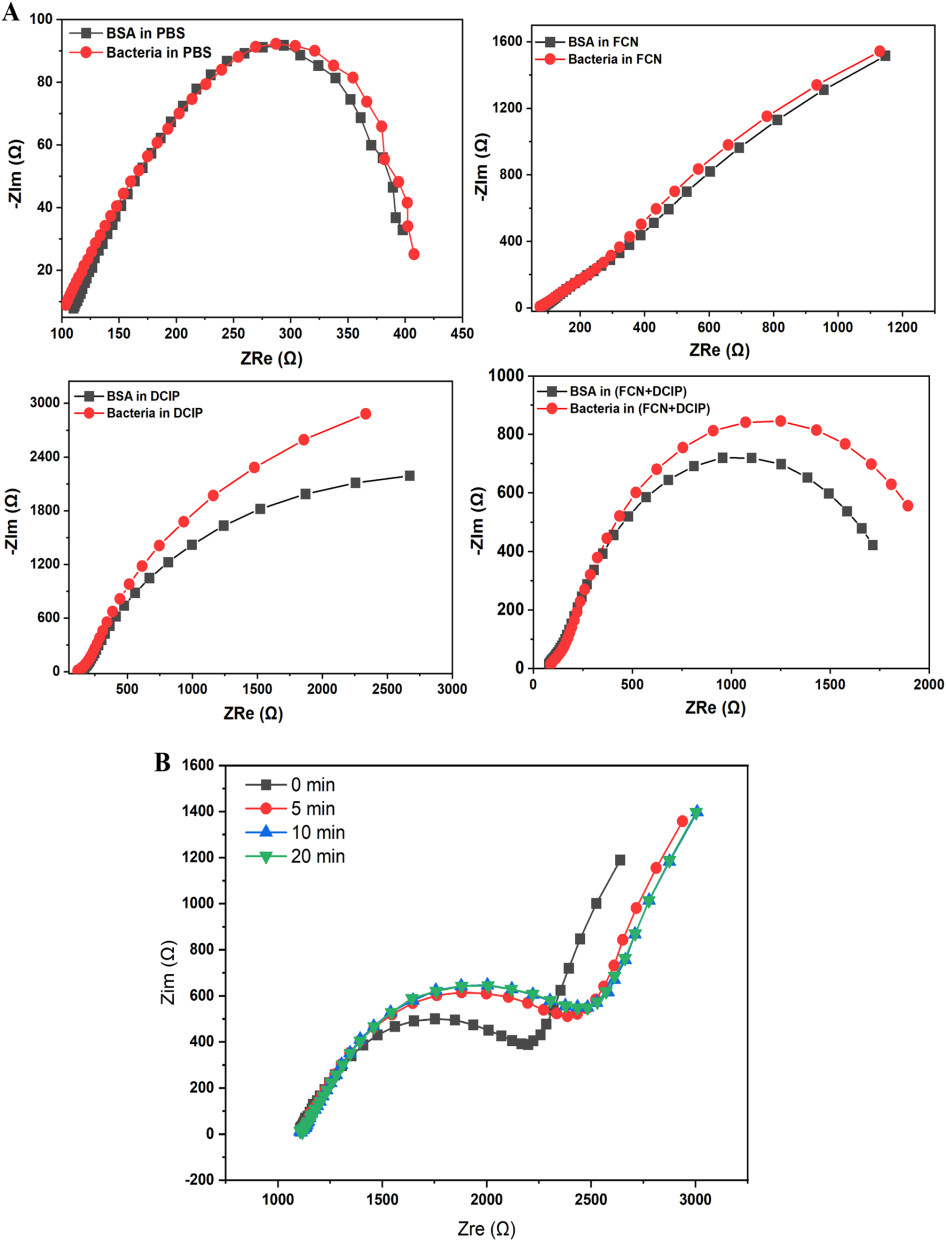
(**A**) EIS Nyquist showing the capturing efficiency of the phage sensors towards the selected pathogen strain. EIS was carried out in different measuring conditions including the sole electrolyte (PBS), FCN, DCIP or a combination of DCIP and FCN. The impedimetric signals were recorded before and after taking place the binding between the immobilized phage and the targeting host cells. (**B**) Effect sensing time on the EIS signal generated by the phage-based biosensor. Bacterial concentration of 1 × 103 CFU/ml was used.

Converting the selective binding interactions between the immobilized phage and its targeting host (*E. coli* O157:H7) into a measurable and quantifiable impedimetric signals was tested in four different conditions. Thus, the capturing efficiency of the phage-sensor to bacterial suspensions was measured in phosphate buffer saline without redox mediators (PBS, as the sole electrolyte), or using a single mediator including FCN, or 2,6-dichlorophenolindophenol (DCIP). Ultimately, a combination of the two mediators (DCIP/FCN) was applied. As a result, the highest impedimetric signal representing the highest sensitivity was obtained when the double mediated system (DCIP/FCN) was used. The use of FCN alone as well as conducting the EIS measurements in PBS without any redox mediator did not show any significant change in the Nyquist plots, as shown in Fig. 3A. Thus, a double mediated system was selected for all further optimizations. The reason behind the EIS signal amplification using the DCIP combined with the FCN is the lipophilicity of the DCIP which enabled this mediator to penetrate the bounded bacterial-phage layers to deliver the redox signal to the final electron acceptor (i.e. the nano-sensor surface).

### Reproducibility, reusability, and sensing time

Five freshly prepared phage-sensor chips were prepared and their EIS signals were collected after the exposure to a single bacterial concentration (10^3^ CFU/ml). As a result, the obtained EIS signals were very close to each other for all tested chips demonstrating the high reproducibility and high accuracy of the fabricated sensor chips. Furthermore, the sensing time (phage-host recognition time) was studied over several time intervals (0, 5, 10 and 20 min). The results (Fig. 3B) showed that 5 min is the minimum needed time for the effective phage-bacteria interaction. This time point (5 min) was selected as the optimal incubation time for conducting the further experiments. Worth mentioning here that the disposable sensor chips cannot be used for several times, thus it can only be used once. This is due to the lytic features of the immobilized phage.

### Calibration curve

**Figure 4:**
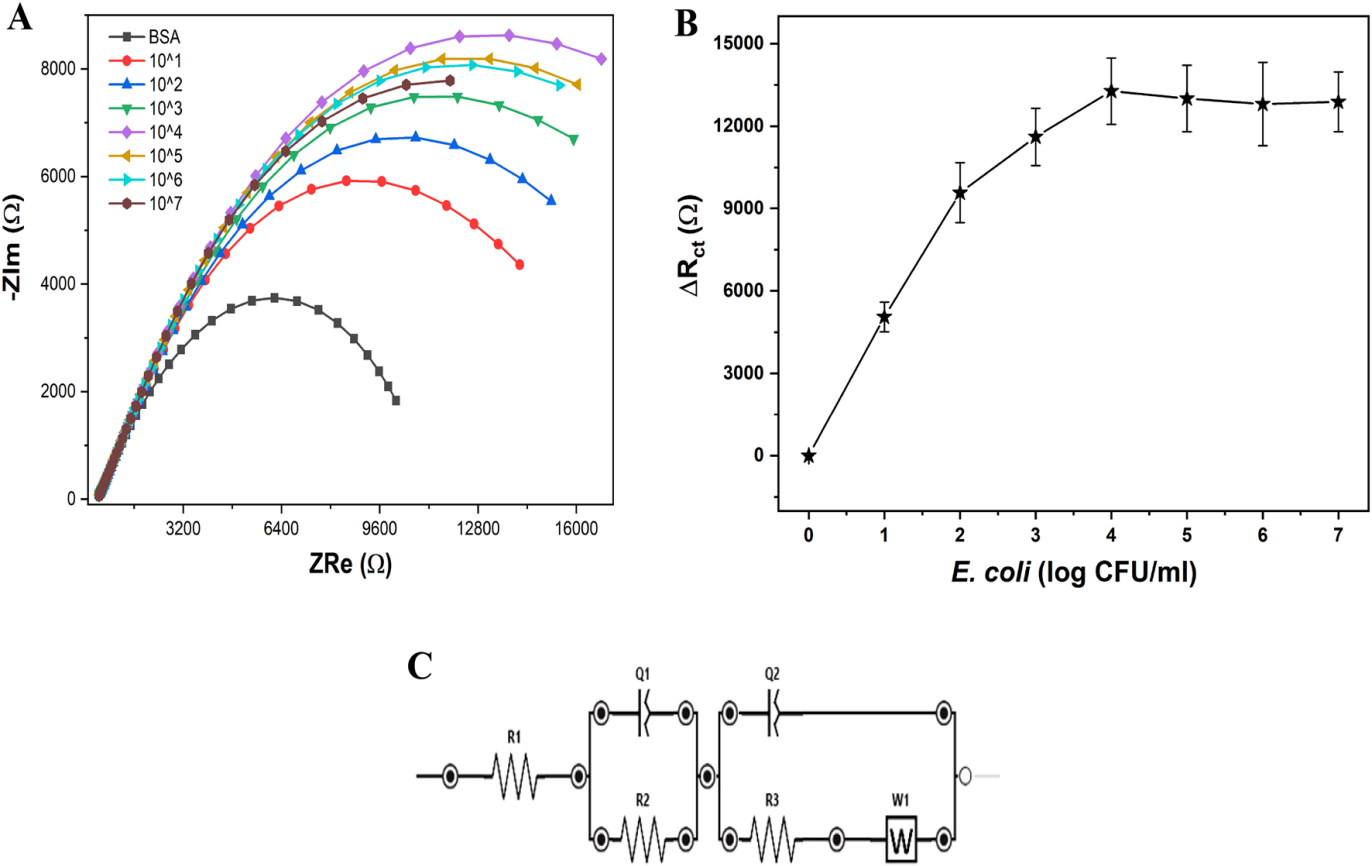
(**A**)-Nyquist plots of the impedimetric calibration curve resulted from the successful binding events taking place at the surface of the phage-based biosensor. Measurements were conducted at different counts of the *E. coli* O157:H7 within the concentration range of 10^1^ to 10^7^ CFU/ml. (**B**) The relationship between the change in the R_ct_ values and the increase in the bacterial counts. (**C**) The Randles equivalent electrical circuit designed for extracting the EIS parameters.

The selective binding efficiency of the phage biosensor was evaluated over a wide range of bacterial concentration (from 10^1^ to 10^7^ CFU/ml), while the EIS spectrum of each concentration was recorded, as it can be depicted from the Nyquist plots, Fig. 4A. A strong correlation between the increase in the bacterial concentration and the increase in the impedimetric signal was obtained and presented in Fig. 4B. To extract R_ct_ values that are demonstrated in this figure, a specific equivalent electrical circuit was modeled (Fig. 4C). The increase in the R_ct_ that resulted from the continuous binding events that are taking place at the sensor surface reached its maximum when the cell concentration reached 10^4^ CFU/ml. At this concentration, a kind of surface saturation level was reached, and this is the maximum capacity for the phage sensor to capture the target host cells. From this investigation, a very high sensitivity was achieved with the limit of detection (LOD) of 3.0 CFU/ml. In Table 1, a collective survey on the performance of other reported impedimetric biosensors for bacterial detection is shown up whereas the highest sensitivity is obtained by the newly developed phage biosensors.

**Table 1:** A collection of the previously reported impedimetric bacterial detections using different types of biorecognition elements and different types of nanomaterials for surface modification.

Tested strain	Surface modification	Method of immobilization	Sensing element	Limit of detection	Ref.
*E. coli* O157:H7	Gold nanoparticle (AuNPs)@gold electrode	EDC/NHS	Antibody	3 × 10^2^—1 × 10^6^ CFU/ml	^31^
*E. coli* O157:H7	Gold	EDC/NHS	Antibody	2 CFU/ml	^32^
*E. coli* O157:H7	Nanoporous membrane of aluminum oxide	Trimethoxysilane-HA-EDC/NHS	Antibody	10 CFU/ml	^33^
*E. coli* O157:H7	Nanoporous membrane of aluminum oxide	Silane-PEG	Antibody	10 CFU/ml	^34^
*E. coli* K-12	Gold microelectrode, interdigitated	Physisorption	T4 bacteriophage	10^4^–10^7^ CFU/ml	^35^
*E. coli* K-12	Microelectrode array Boron-doped UNCD	Physisorption	Antibody	NA	^36^
*E. coli* O157:H7	Interdigitated microelectrode	Physisorption	Antibody	2.5 × 10^4^—2.5 × 10^7^ CFU/ml	^37^
*E. coli*	Gold	SAM-EDC/NHS	Antibody	1.0 × 10^3^ CFU/m	^38^
*E. coli*	Gold	SAM-biotin-NeutrAvidin	Biotinyl antibody	10 CFU/ml	^39^
*E. coli*	Tungsten- gold- wire	Polyethyleneimine-streptavidin	Biotinyl antibody	10^3^–10^8^ CFU/ml	^40^
*E. coli*	Gold disk	mSAM	Synthetic glycan	10^2^–10^3^ CFU/ml	^41^
*E. coli*	Interdigitated polysilicon electrodes	Glutaraldehyde	Antibody	3 × 10^2^ CFU/ml	^42^
*E. coli* O157:H7	Gold	SAM-HA-EDC/NHS	Antibody	7 CFU/ml	^43^
*E. coli*	Gold	SAM-PDICT cross-linker	Bacteriophage	8 × 10^2^CFU/ml	^44^
*E. coli*	Paper of graphene	Biotin-streptavidin	Antibody	1.5 × 10^2^ CFU/ml	^45^
*E. coli*	A microarray of printed electrode	EDC/NHS	Bacteriophage	10^4^ CFU/ml for 50-ul samples	^46^
Sulfate-reducing bacteria	GCE	Reduced graphene sheet with chitosan plus 1% glutaraldehyde	Antibody	1.8 × 10^1^–1.8 10^7^ CFU/ml	^47^
Sulfate-reducing bacteria	ITO	Chitosan-reduced graphene sheet	Bacterial imprinting	1.0 × 10^4^–1.0 × 10^8^ CFU/ml	^48^
Sulfate-reducing bacteria	Ni-foam	Nanoparticle-SAM-EDC/NHS	Antibody	2.1 × 10^1^–2.1 × 10^7^ CFU/ml	^49^
*Salmonella Typhimurium*	Flat gold	SAM-glutaraldehyde	Antibody	NA	^50^
*Salmonella Typhimurium*	Deposited gold film/SPE	16-MHDA-EDC-NHS	Monoclonal antibody	10 CFU in 100 ml	^51^
*Salmonella Typhimurium*	Gold	Polytyramine-glutaraldehyde	Antibody	NA	^50^
*Campylobacter jejuni*	Glassy carbon	Physisorbed onto *O*carboxymethylchitosan surface-modified Fe3O4 nanoparticles	Monoclonal antibody	1.0 × 10^3^–1.0 × 10^7^ CFU/ml	^52^
*Listeria innocua*	Gold	SAM-EDC/NHS	Endolysin (bacteriophage encoded peptidoglycan hydrolases)	1.1 × 10^4^ and 10^5^ CFU/ml	^53^
*Staphylococcus aureus*	Nanoporous alumina	Silane (1%) GPMS	Antibody	10^2^ CFU/ml	^54^
*Porphyromonas gingivalis, E. coli*	Microfluidic cell with hydrodynamic focusing	No immobilization/impedance reading during flow of cells	None	1.0 × 10^3^ cells/ml	^55^
*E. coli* O157:H7	Au/WO_3_/MWCNTs nano-composite/SPEs	Self-assembled monolayers (SAMs) of 4-Aminothiophenol (4-ATP) and glutaraldehyde	ZCEC5 phage	3 CFU/ml	***

(***) is the newly developed phage biosensor.

### Selectivity testing

**Figure 5:**
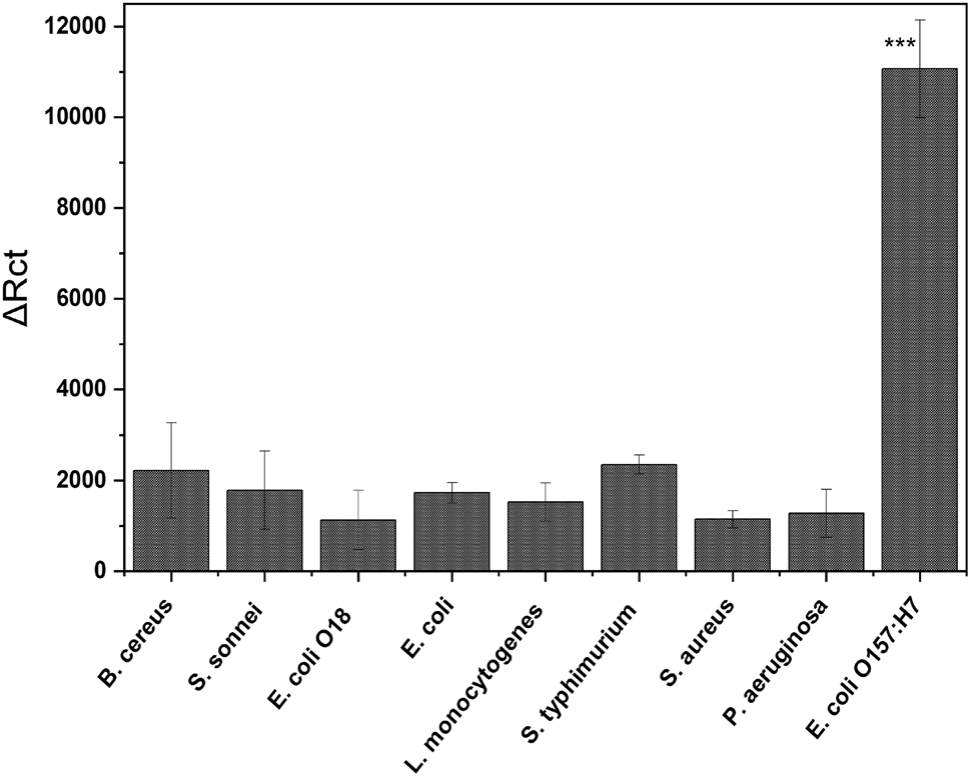
The impedimetric responses of the phage-based biosensor towards the targeting (***) and non-targeting bacterial strains.

Since the selection of a biorecognition element(s) is always necessary for offering the maximum selectivity and specificity, the bio-sensing performance of the newly developed phage-based biosensor was tested against many other bacterial strains, as shown in Fig. 5. As a result, high selectivity was obtained whereas non-significant responses were found when the foreign bacterial strains were tested. Thus, the selection of this bacteriophage as a bio-recognition element offered a rapid, selective and quantitative impedimetric detection for the targeting organism.

### Analysis of artificial food samples

To ensure that the phage-biosensor is ready to use for sample analysis, synthetic bacterial contamination (spiking the food samples with *E. coli* O157:H7 at the concentration of 10^3^ CFU/ml) for a variety of food samples including beef meat, white cheese, tomato juice, tap water, and luncheon beef meat samples are tested. Meanwhile non-infected (not-spiked) samples were also included in that test as a negative control. For each sample, an individual sensor chip was used, and the change in the ΔR_ct_ was calculated. In parallel to that test, CFU plate counting was conducted for the validation. As a result, a recovery of 90–100% has been obtained (Table 2).

**Table 2:** Food sample analysis and recovery percentage of bacterial contaminations.

	ΔR_ct_ (Ohm)	Recovery (%)
*E. coli* standard suspension	3433	100
Spiked beef meat	3224	94
Spiked white cheese	3512	102
Spiked tab water	2845	83
Spiked tomato juice	3169	92
Spiked luncheon meat	3061	89
(-)-Control tab water	921	27
(-)-Control tomato juice	386	11
(-)-Control luncheon meat	746	22

## Conclusion

With the continuous emergence of highly contagious strains and the increase of bacterial infections, development of electrochemical nan-biosensors is usually utilized for single-use tests to afford the fast onsite detection, and to avoid sensor cleaning procedures or cross-contaminations. Here, disposable screen printed electrodes were functionalized with a nanocomposite which consisted of gold, tungsten oxide and carbon nanotubes (Au/WO_3_/MWCNTs) to provide high electrocatalytic and electrochemical readouts. As a biorecognition element, a lytic bacteriophage (*E. coli* T4-like virus) was covalently immobilized onto the nanosensor’s surface that was cross-linked with self-assembled monolayers (SAMs) of 4-Aminothiophenol (4-ATP) and glutaraldehyde. Voltammetric as well as impedimetric methods were used for the electrochemical characterizations, while the assay optimization and real sample analysis were completed with the impedimetric method. With high sensitivity and selectivity provided by the phage-based biosensor, food sample analysis was successfully conducted. Thus, this biosensor is very promising and opens a new avenue towards the pathogen diagnosis using disposable and onsite devices.

## Methods

### Bacterial culture and bacteriophage preparation

As previously described^56^, the targeting phage implemented in this study (ZCEC5, T4 like the wild type) has been isolated from environmental samples. The ZCEC5 phage genome sequence is existing in GenBank under the Accession Number MK-542015. *E. coli* O157:H7 (NCTC-12900) was selected as the sole bacterial host for the phage propagation and amplification. For the selectivity and interference study, the following bacterial strains have been: *Staphylococcus aureus* (ATCC-25923), *Salmonella typhimurium* (ATCC-14028), *Listeria monocytogenes* (ATCC-13932), *E. coli* (ATCC-8739), *Bacillus cereus (*ATCC-11778), and *Shigella sonnei* (NCTC-12984, ATCC-29930) were provided by the American Type Culture Collection (Manassas, VA, USA). *E. coli-* O18, Accession No. OK355402 was provided by MEVAC Company, Egypt. *Pseudomonas aeruginosa* is the local lab isolate (under submission to Genbank). All bacterial strains were cultivated in Luria–Bertani (LB) broth for overnight at 37 °C with shaking at 120 RPM. Cell pellets of overnight cultures were collected by centrifugation at 5000 rpm for 10 min. Pellets were thoroughly washed and re-suspended in PBS. The bacterial count was performed using plate-count techniques and expressed in CFU/ml. Phage ZCEC5 titer was determined by double-agar overlay plaque assay techniques and expressed in PFU/ml as well. Briefly, 200 μl of mid-log host bacterial culture was mixed with 100 μl of serially diluted bacteriophage suspension, and 5 ml of LB top agar (0.7%) was poured on to a LB agar base plate (1.5%) and incubated overnight at 37 °C^57^.

### Sensors platform modification with nanomaterials

Electrochemical measurements were carried out using a computer-controlled Palmsens-4 Potentiostat/Galvanostat/Impedance Analyzer. As a phage-biosensor platform, disposable screen-printed carbon electrodes (SPEs) were used. The working area of the SPE was individually modified with each of the selected nanomaterials including gold nanoparticles (AuNPs), multi-walled carbon nanotubes (MWCNTs) and nanostructured tungsten(VI) oxide (WO_3_). The nanomaterials were drop-casted onto the 3.0 mm active working electrode of plain carbon screen-printed electrodes. In Addition, composites of these nanomaterials were prepared and their electrochemical characteristics were identified. In this regard, cyclic voltammetry (CV) was carried out at a scan rate of 50 mV/s, and an electric potential ranging from −0.4 to 0.7 V vs the Ag/AgCl was applied. Electrochemical impedance spectroscopy (EIS) was conducted at AC potential of 5 mV and the applied frequency sweeps extended from 10,000 to 0.1 Hz. As a redox probe, 5 mM of potassium ferricyanide (III) (FCN, Merck, USA) was used for the electrochemical characterizations using the cyclic voltammetry (CV), and electrochemical impedance spectroscopy (EIS). From the nanomaterial screening step, WO_3_/MWCNTs nanocomposite at a ratio of (2:1 v/v) was selected and employed as the sensor platform for the electrochemical biosensing applications. To evaluate the individual resistance of the electrochemical system, impedance data were fitted to an equivalent circuit (Randles) model. All the electrochemical tests were conducted at room temperature (25 ± 2 °C). The chemical functionalization was investigated by the Fourier Transform Infrared (FTIR) spectroscopy (Thermo Scientific Nicolet iS10 FT-IR).

### Phage immobilization on the nanostructured sensor chips

To provide an effective method for the bacteriophage immobilization on the nano-structured electrode, a two-step process for the self-assembled monolayer (SAM) formation using 4-aminothiophenol (4-ATP), and glutaraldehyde (Glu) was applied, respectively^14,58^. Firstly, the modified SPEs with the nanocomposite (AuNPs/WO_3_/MWCNTs) were incubated for 12 h in a solution of 4-ATP (50 mM) at 4 °C. Subsequently, the electrode chips were washed thoroughly with ethanol to remove the unbounded thiols from the surface. Subsequently, the self-assembled 4-ATP monolayer formed on the chip surface is then activated by the 2% of glutaraldehyde solution (C_5_H_8_O_2_) for 1 h. After the formation of the aldehyde group on the sensor chips, the surfaces were washed twice with deionized water, and dried before they incubated with the active T4 wild type phage (ZCEC5) (titer 10^9^) for 20 h, at 40 °C. Eventually, the prepared phage-based biosensor was washed and immersed in BSA (1 mg/ml) for 30 min at room temperature. Figure 1 demonstrated the fabrication steps of the proposed phage-based biosensor.

### Testing the biosensors performance using a double mediated electron transfer system

The performance of the prepared biosensor was tested against the targeting bacterial strain (*E. coli* O157:H7) in the electrochemical cell with or without redox mediators. In this experiment, two different types of redox mediators (ferricyanide (FCN, 5 mM) as a hydrophilic mediator, and 2,6-dichlorophenolindophenol (DCIP, 40 μM) as a lipophilic mediator) were exploited for the impedimetric signal amplification^21^. Each of these mediators was tested separately, before mixing them together in another experiment. EIS experiments were carried out and the biosensor performance was evaluated. As a negative control, a non-mediated system was used whereas the biosensor was assessed in the supporting electrolyte (PBS) without the use of redox mediators.

### Calibration curve, and sensitivity testing

After preparing a serial dilution from the *E. coli* O157:H7 bacterium (from 10^1^ to 10^7^ CFU/ml) in phosphate buffer, the biosensor chip was incubated with each concentration for 5 min, then washed, before mounting the chip into the electrochemical cells to record the generated EIS signal. For each bacterial count, EIS was measured before and after exposing the sensor chip to the bacterial concentration. Then the change in charge transfer resistance (ΔR_ct_) was calculated according to a modeled equivalent Randles-circuit.

### Selectivity and interference

Responses of the phage biosensor to foreign (non-targeting) bacterial strains were tested here to evaluate the degree of cross-reactivity. Therefore, a suspension of 10^3^ CFU/ml of each of non-targeting foodborne pathogens including *Bacillus cereus*, *Shigella sonnei*, *Listeria monocytogenes*, *Salmonella typhimurium*, *Staphylococcus aureus*, *Pseudomonas aeruginosa*, *E. coli* O18, *E. coli*-ATCC 8739, and *E. coli* 157: H7 NCTC 12,900. A separate incubation between the biosensor chips and each of the prepared suspension for 5 min took place before measuring the EIS responses. The EIS of the biosensor's response towards the targeting strain was added as a positive control.

### Food sample analysis

For practical application and validation, the efficiency of the biosensor was evaluated on several synthetic infected food samples (beef meat, white cheese, tap water, tomato juice, and luncheon beef meat). In addition, three non-contaminated samples (tap water, tomato juice, and luncheon meat) were used as a negative control. For instance, each of these samples was spiked and homogenized with a certain suspension of the *E. coli* O157:H7 (10^3^ CFU/ml). For each food sample, a freshly prepared biosensor’s chip was incubated for 5 min, and then the EIS data was evaluated.

### Statistics and data analysis

All data are presented as the mean ± SD from at least three individual experiments. Statistical significance was determined by statistical hypothesis testing where the significance of the values was estimated as *p* < 0.05. From the standard calibration curves, the limit of detection (LOD) was calculated. The reproducibility of the phage biosensor performance was represented by the relative standard deviation (RSD). All the statistical, and data analysis was performed using Origin Lab software which was used for drawing all the presented figures.

## Supplementary Information

Supplementary Information.

## Data Availability

Data supporting the findings of this study are available within the article.
