# Supplementary material for: Bacteriophage-based nano-biosensors for the fast impedimetric determination of pathogens in food samples
Source: Sci Rep. 2023 Mar 1;13:3498. doi: 10.1038/s41598-023-30520-3 (PMC9977096; doi:10.1038/s41598-023-30520-3)
Supplement: Supplementary file 1 — Supplementary Information. [file 41598_2023_30520_MOESM1_ESM.doc]

**Bacteriophage-Based Nano-biosensors for the Fast Impedimetric Determination of Pathogens in Food Samples**

Nader Abdelhameed^1^, Fatma [Abdelrahman](https://sciprofiles.com/profile/1397970)^2^, Ayman [El-Shibiny](https://sciprofiles.com/profile/1212554)^2^*, and Rabeay Y. A. Hassan^1^*

^1^Nanoscience Program, University of Science and Technology (UST), Zewail City of Science and Technology, Giza 12578, Egypt

^2^Center for Microbiology and Phage Therapy, Biomedical Sciences, Zewail City of Science and Technology, Giza 12578, Egypt

***Corresponding Authors:**

**1-Rabeay Y. A. Hassan**

Nanoscience Program, University of Science and Technology (UST),

Zewail City of Science and Technology,

6th October City, 12578 Giza, Egypt

Email: [ryounes@zewailcity.edu.eg](mailto:ryounes@zewailcity.edu.eg)

**2-Ayman** [**El-Shibiny**](https://sciprofiles.com/profile/1212554)

Center for Microbiology and Phage Therapy,

Biomedical Sciences, Zewail City of Science and Technology,

Giza 12578, Egypt

***Selectivity testing***

**Table S1:** testing the selectivity performance of the phage-based biosensor towards several foreigner bacterial strains.

| **Bacterial strains** | ***ΔR_ct_*** (Ohm) | **±SD** (Ohm) |
| --- | --- | --- |
| B. cereus | 2221,6 | 1049 |
| *S. sonnei* | 1789,3 | 863 |
| *E. coli O18* | 1128,4 | 658 |
| *E. coli* | 1732,8 | 227 |
| *L. monocytogenes* | 1529 | 422 |
| *S. typhimurium* | 2354,3 | 213 |
| *S. aureus* | 1147,3 | 191 |
| *P. aeruginosa* | 1280,9 | 529 |
| *E. coli O157:H7 (the target organisms)* | 11067,6 | 1075 |

**Real food sample analysis**

Application of the newly developed phage-based biosensors for real food sample analysis

**Table S2:** Food sample analysis and recovery percentage of bacterial contaminations

|  | ΔR_ct_ (Ohm) | Recovery (%) |
| --- | --- | --- |
| *E. coli* standard suspension | 3433 | 100 |
| Spiked beef meat | 3224 | 94 |
| Spiked white cheese | 3512 | 102 |
| Spiked tab water | 2845 | 83 |
| Spiked tomato juice | 3169 | 92 |
| Spiked luncheon meat | 3061 | 89 |
| (-)-Control tab water | 921 | 27 |
| (-)-Control tomato juice | 386 | 11 |
| (-)-Control luncheon meat | 746 | 22 |
